# Nitrofurantoin treatment and prophylaxis for recurrent urinary tract infections in women: First-line for a reason

**DOI:** 10.1371/journal.ppat.1014516

**Published:** 2026-08-13

**Authors:** Alec Szlachta-McGinn, A. Lenore Ackerman

**Affiliations:** 1 Department of Obstetrics and Gynecology, Division of Urogynecology and Reconstructive Pelvic Surgery, Washington University in St. Louis, St. Louis, Missouri, United States of America; 2 Department of Urology, Division of Urogynecology and Reconstructive Pelvic Surgery, David Geffen School of Medicine, University of California Los Angeles, Los Angeles, California, United States of America; Tufts Univ School of Medicine, UNITED STATES OF AMERICA

## Abstract

Nitrofurantoin is considered a first-line antimicrobial agent by the AUA/CUA/SUFU Guideline and Infectious Disease Society of America (IDSA) Guideline for the treatment of acute uncomplicated simple cystitis in women. Nitrofurantoin has good oral bioavailability, high concentration in urine, and low serum and tissue concentrations. These characteristics result in minimal collateral damage to the microbial composition of other niches within the human body, such as the gut, that are essential in maintaining homeostasis. In addition, resistance to nitrofurantoin is uncommon owing to its interference with multiple bacterial cellular functions critical to survival. Large systematic reviews and meta-analyses have demonstrated that nitrofurantoin is noninferior to other commonly prescribed antibiotics for treatment of urinary tract infection (UTI). Recent data from a randomized controlled trial suggest that a 5-day course of nitrofurantoin may be more effective than a single dose of fosfomycin in the treatment of UTI. Therefore, nitrofurantoin is an ideal first-line agent in the management of UTI.

## Introduction

Recurrent UTI is defined in the American Urologic Association (AUA) Guideline for the evaluation and management of recurrent urinary tract infection (UTI) as two culture-proven episodes of acute bacterial cystitis within six months or three episodes within 1 year [1]. Approximately 50–70% of women will experience UTI in their lifetime, and approximately one-quarter of those will subsequently develop recurrent infections [1]. Cranberry, D-mannose, methenamine hippurate, probiotics, and antibiotics at reduced doses are used as prophylactic interventions to prevent recurrent UTI with various evidence for success [1]. While multiple specialty society guidelines exist for the management of UTI, often differing on the fine details of these approaches, broad consensus exists for the use of nitrofurantoin as first-line antibiotic treatment for acute bacterial cystitis in women, due to the high efficacy, low rates of resistance, and infrequency of serious side effects [1,2]. Despite this consensus, nitrofurantoin remains underutilized for UTI treatment and prevention. Unfamiliarity with current guidelines and the unique benefits and low side effect profile of nitrofurantoin are likely reasons for its underuse, though this remains understudied in the literature. This article seeks to review and evaluate the current evidence to support the use of nitrofurantoin for the management and prevention of acute, uncomplicated bacterial cystitis, particularly in women with recurrent UTI. The ensuing discussion excludes the following special populations: pregnant women; those who are immunocompromised; those with functional or anatomic abnormalities of the urinary tract; and those with indwelling catheters or who self-catheterize.

## 1. Nitrofurantoin exhibits potent activity in the urine without substantial systemic impact

Nitrofurantoin, absorbed predominantly in the small intestine with 80% oral bioavailability, is renally cleared to result in therapeutic concentrations in the urine rapidly after ingestion [3]. Therapeutic drug levels are not achieved in serum; poor tissue penetrance precludes its use for infections other than bacterial cystitis [4]. Bacterial nitroreductases convert nitrofurantoin to active metabolites that interfere with DNA, RNA, protein, and cell wall synthesis, giving nitrofurantoin bactericidal properties in the lower urinary tract [5]. As the active metabolites of nitrofurantoin interfere with bacterial cell homeostasis at multiple checkpoints, drug resistance is uncommon [6], although rare mutations in nitroreductase genes in *E. coli* confer nitrofurantoin resistance [7]. Nitrofurantoin has activity against both gram-positive and gram-negative microbes, including most UTI-causing pathogens such as *E. coli* and *Enterococcus* species [3].

## 2. Nitrofurantoin exhibits few side effects and low propensity for collateral damage

While commonly prescribed antibiotics, including nitrofurantoin, TMP-SMX, fosfomycin, beta-lactams, and fluoroquinolones, all have ~90% efficacy in treating UTIs, each is associated with different side effects and propensities for “collateral damage” [2]. While all antibiotics can induce gastrointestinal distress with nausea, vomiting, and diarrhea, these side effects are less common with nitrofurantoin than with other antibiotics [2]. Rare reports of pulmonary and hepatoxicity have been linked to nitrofurantoin, however most of these serious adverse events are associated with chronic use [8]. While nitrofurantoin-associated pulmonary toxicity is also perpetuated as a reason to avoid its use, in reality, this complication is rare; most cases are reported in patients receiving extended therapy for longer than the 5-day course recommended as treatment for cystitis [2].

Collateral damage refers to the “ecological adverse effects of antimicrobial therapy,” such as the selection of drug-resistant organisms and the development of colonization or infection with multidrug-resistant organisms [9]. These effects are thought to result from disruption of the normal human microbial communities (“dysbiosis”) after antibiotic exposure, with the impacts on the gut microbiome being the best studied [2,10]. Gut dysbiosis induced by antibiotics can both directly enrich for uropathogenic overgrowth and can induce systemic alterations in inflammation, tissue permeability, and metabolism that also decrease host resistance to infection recurrence. Beta-lactams and fluoroquinolones have the highest propensity for collateral damage compared with nitrofurantoin, fosfomycin, and TMP-SMX [9], resulting in both short- and long-term alterations in the gut microbiota, including overall reduced diversity and richness and decreased levels of *Lactobacillus*, *Bacteroides*, and *Bifidobacterium* species [10]. Culture-independent sequencing of fecal samples before and after antibiotics use demonstrates that fluoroquinolones induce a greater global dysbiosis of the gut microbiome relative to nitrofurantoin [11]. Nitrofurantoin has the lowest risk of gut dysbiosis compared with fosfomycin or TMP-SMX use [2]. There is a lack of data on collateral damage to the vaginal and urinary microbiomes with antibiotic use.

## 3. Antimicrobial resistance associated with nitrofurantoin is rare

Nitrofurantoin has significantly lower antimicrobial resistance rates than other antibiotics [6]. A study of over 12 million *in vitro* antimicrobial resistance assays of urinary *E. coli* isolates from U.S. outpatients between 2000 and 2010 demonstrated large increases in resistance to ciprofloxacin (3%–17.1%) and TMP-SMX (17.9%–24.2%) with minimal increase in resistance to nitrofurantoin (0.8%–1.6%) [12]. A systematic review of *E. coli* resistance patterns for uncomplicated acute cystitis found an increase in fluoroquinolone resistance worldwide between 2008 and 2017 with estimates of ~12% in North America but as high as 40% in other countries [13]. Additionally, fluoroquinolone use is associated with an increased risk of infection with methicillin-resistant *Staphylococcus aureus* and fluoroquinolone-resistant *Pseudomonas* [9]. Cephalosporin use is associated with an increased risk of infection with vancomycin-resistant *Enterococcus* species, extended spectrum beta-lactamase producing *Klebsiella* and *Acinetobacter* species, and *Clostridium difficile*.[9]

## 4. Due to its localized action, there are few contraindications to nitrofurantoin use

As with all medications, nitrofurantoin should not be administered to those with a hypersensitivity or allergic reaction to this medication [4]. Due to its rapid clearance from serum, nitrofurantoin cannot treat the bacteremia associated with systemic infections, such as pyelonephritis or urosepsis [4]. Thus, nitrofurantoin should not be given for the treatment of pyelonephritis, as nitrofurantoin does not accumulate sufficiently in any tissue, with the exception of the urine. Fear of progressive infections may partially explain the underutilization of nitrofurantoin; however, progression from uncomplicated UTI to pyelonephritis is rare with annual rates between 2 and 3 cases per 10,000 of uncomplicated UTI [1].

Prior literature has cautioned against its use in those with creatinine clearance of less than 60 milliliters per second (mL/s), which remains on the manufacturer labeling [4]. Newer, retrospective data, however, have demonstrated that nitrofurantoin is safe in patients with creatinine clearance greater than 30 mL/s, guidance echoed in the American Geriatrics Society 2015 Updated Beers Criteria [14].

## 5. Nitrofurantoin is effective in the management and prevention of recurrent UTI

Nitrofurantoin is a first-line recommended antibiotic for the management of acute, uncomplicated cystitis; other first-line agents include fosfomycin and TMP-SMX [1,2]. Several large meta-analyses and Cochrane reviews have demonstrated the noninferiority of nitrofurantoin to TMP-SMX, beta-lactams, and fluoroquinolones in clinical and microbiological cure rates [6,15].

An analyst-blinded, randomized controlled trial published in 2015 comparing 5 days of nitrofurantoin versus a single dose of fosfomycin for the management of acute uncomplicated cystitis in 513 women demonstrated superior efficacy of nitrofurantoin (100 milligrams (mg) orally three times daily) compared to fosfomycin (single 3 gram (g) dose) in clinical (70% versus 58%, respectively, *p* = 0.004) and microbiological cure rates (74% versus 63% respectively, *p* = 0.04) with no difference in adverse events [16]. A subgroup analysis of infection with *E. coli* demonstrated greater cure rates with nitrofurantoin versus fosfomycin (78% versus 50%, respectively, *p* < 0.001) [16]. This is in contrast to two previous double-blinded, randomized clinical trials published in the 1990s, including almost 1,000 women together that demonstrated equivalent cure rates [17,18].

A systematic review and meta-analysis of nitrofurantoin use for UTI prophylaxis of 3,052 patients found that nitrofurantoin is superior to placebo in the prevention of UTI and comparable to other antibiotics [19]. While the meta-analysis also found an elevated risk of nonsevere adverse events (nausea, vomiting, pruritus, and headache) with prolonged nitrofurantoin use, the risk of severe toxicity was rare and related to chronicity of use [19]. A cohort study of 1,893 women using nitrofurantoin prophylaxis compared 50–100 mg daily dosing and demonstrated equivalent hazard of UTI and pyelonephritis; however, 100 mg dosing was associated with greater risk of cough, dyspnea, and nausea [20]. Thus, some of the mild adverse events may be averted with use of a lower dose for daily prophylaxis.

## Conclusions

In summary, nitrofurantoin has good efficacy and safety data in the management of acute uncomplicated cystitis in women for its use as a first-line antimicrobial agent in the management of UTI with culture-proven susceptibility. Compared to the other available first-line antibiotics in the treatment of UTI, nitrofurantoin may be superior to a single dose of fosfomycin and is associated with less adverse events and gastrointestinal collateral damage than TMP-SMX with no difference in clinical efficacy. Beta-lactams and ciprofloxacin are associated with significant potential for serious collateral damage and should not be used as first-line agents. While there is relatively limited data guiding nitrofurantoin use in women with recurrent UTI, considerations for maximizing antimicrobial efficacy while minimizing collateral damage are especially important in women who may receive multiple courses of antibiotics per year for treatment of UTI. Therefore, despite the limited evidence, nitrofurantoin should be used as a first-line agent for recurrent UTI in women.
